# Saudi dental students’ perceptions on sustainable development goals and sustainable dental practice

**DOI:** 10.1038/s41405-024-00228-1

**Published:** 2024-05-30

**Authors:** Sanjida Haque, Mohammad Nurunnabi, Tahsinul Haque

**Affiliations:** 1 Center of Excellence in Cyber-Security; Center for Sustainability and Climate, Prince Sultan University, Riyadh, Saudi Arabia; 2 Center for Sustainability and Climate, Prince Sultan University, Riyadh, Saudi Arabia; 3 Department of Preventive Dental Sciences, College of Dentistry, Dar Al Uloom University, Riyadh, Saudi Arabia

**Keywords:** Dental education, Dental clinical teaching

## Abstract

**Objectives:**

This study utilised a cross-sectional survey design to assess the levels of knowledge and awareness among 841 undergraduate dental students from Saudi Arabia regarding sustainable development goals (SDGs) and sustainable dental practices (SDP).

**Materials and methods:**

A self-administered online questionnaire was distributed to adults aged between 18 and 35 years of both genders, from November 2022 to November 2023. The study has obtained appropriate ethical approval.

**Results:**

Participants exhibited a moderate level of knowledge and awareness regarding the SDGs, while demonstrating a high level of awareness specifically related to the SDP. The presence of a correlation between gender-associated beliefs and pro-environmental behaviours is apparent. Additionally, it has been observed that participants who engage in clinical activities exhibit a heightened level of awareness concerning SDP.

**Conclusions:**

By assessing dental students’ current knowledge and awareness of the SDGs and SDP, we can inform stakeholders in the dental industry about how to enhance sustainability competence and develop dental policy curricula. This will better prepare students to serve as educators as well as professionals, aligning with their commitment to integrating the principles and objectives of various SDGs and SDP into dental education and practice.

## Introduction

The growing prominence of climate change has led to a noticeable increase in human awareness and concern regarding ecological issues. The current emphasis has transitioned from the sole pursuit of development to the imperative of mitigating our environmental footprint and promoting sustainable development [1]. The concept of sustainable development places emphasis on the integration of development activities with the preservation and responsible utilisation of natural resources, with the ultimate goal of meeting the needs and aspirations of future generations [2].

The incorporation of this concept within the field of dentistry is a viable approach, as the existing model of dental care delivery in contemporary society faces challenges that hinder its long-term sustainability. These challenges include escalating financial expenses, growing demands, and environmental concerns [3, 4]. The prioritisation of efficiency by dental professionals often results in the production of significant amounts of waste and carbon dioxide emissions, thereby indicating a potential lack of emphasis on sustainability within the industry. Research has found that dental practices dispose of a significant number of sterilisation pouches, chair barriers, and light handle covers every year [5]. This accumulation of waste poses a considerable environmental concern and calls for effective waste management strategies within the dental industry. The breakdown of gloves, masks, suction tips, saliva ejectors, needles, and paper, along with other disposable items, poses challenges. Literature also observes a staggering daily waste of 360 gallons of water. Furthermore, on an annual basis, the generation of 4.8 million lead foils, approximately 28 million litres of poisonous X-ray fixer, and a significant 3.7 tonnes of mercury waste is causing alarm. The concerning scale of waste being produced necessitates urgent attention and effective management strategies, as highlighted by these figures [3, 5–7].

The United Nations has established the 17 sustainable development goals (SDGs) as a comprehensive framework to advance, in a sustainable manner, global peace, prosperity, and also wellbeing. These goals represent a collaborative endeavour aimed at addressing various social, economic, and environmental challenges faced by societies worldwide. By pursuing these goals, the international community seeks to ensure a harmonious and enduring future for all [8].

The potential impacts of each one of these SDGs upon oral health are worth exploring. For instance, a potential approach to mitigating the issue of unequal access to oral health care is by addressing poverty, as outlined in SDG 1. By focusing on poverty reduction, it is possible to alleviate the disparities in accessing oral health care services. Addressing the issue of hunger in alignment with SDG 2 has the potential to significantly mitigate the adverse effects of malnutrition on oral health. In order to address gender and social inequality, it is important to focus on the attainment of SDG 5 and SDG 6. By achieving these goals, it is expected that gender discrimination within the labour market will be lessened, leading to positive outcomes such as improved maternal education and increased economic opportunities. These factors play crucial roles in the prevention and control of early childhood caries. Moreover, it is worth noting that SDG 9 emphasises the importance of implementing inventive approaches, establishing infrastructure that facilitates growth, and encouraging industry investment in emerging technologies. These aspects hold significant relevance in the context of developing novel non-invasive methods for the control of caries. The intricate dynamics between economic, social, and environmental elements within urban areas, as exemplified by SDG 11, present noteworthy risk factors for caries. It is observed that the prevalence of caries and inadequate periodontal health is elevated in slum areas. In contrast, it is worth noting that prioritising oral health has the potential to make a substantial impact on the achievement of the SDGs. One potential approach to contribute to the achievement of SDG 12 is by placing emphasis on preventive measures, the promotion of oral health, and the utilisation of recyclable dental materials. By adopting these strategies, it is possible to align with the goals outlined in SDG 12. As a result, improving oral health on a global scale is crucial to accomplishing the SDGs [4, 9–12].

The concept of sustainable dental practices (SDP) revolves around the promotion and integration of sustainability principles within the field of dental practice. The terms “environmental sustainability in dentistry”, “green dentistry”, “sustainable dental practices”, and “eco-friendly dentistry” are often employed interchangeably to refer to similar concepts within the field. In 2007, the concept of eco-friendly dentistry was introduced by Farahani and Suchak, marking a significant milestone in the field. The green model proposed in their research focuses on the implementation of eco-friendly alternatives to conventional dental practices [13]. It aims to replace the harmful current dental practices with environmentally sustainable alternatives. The process of transitioning from a traditional dental practice to an SDP in order to promote environmental sustainability has been found to potentially require significant time and financial investments [14]. However, it is important to note that such a transition is indeed practicable and has been successfully implemented in various countries [15, 16]. In recent years, there has been a growing global effort among dental professionals, stakeholders, and policymakers to mitigate the environmental consequences associated with dental practice. This collaborative endeavour aims to enhance the sustainability of daily clinical routines by implementing ecological practices. The integration of sustainable development objectives into clinical practice within the field of dentistry is crucial in order to contribute to the overall transition towards an eco-friendly economy. This can be achieved through the implementation of SDP [14–16].

Since oral health is a crucial part of human life, dental professionals should incorporate SDGs as well as sustainable dental practices (SDP) into their daily lives, raise awareness of their environment, and encourage the transition to a green economy at all stages of life. Every professional in the field of dentistry should have sufficient knowledge about SDGs as well as SDP and regulate their procedures while taking sustainability principles into account. The World Federation of Dentistry (FDI) also called for sustainable techniques in oral health care to safeguard service delivery and foster a green economy [17].

Dental students, as future dental professionals, will play crucial roles in addressing SDGs and SDP and fostering community unity. It is essential for them to possess sufficient knowledge and a positive perception in order to bridge the gap and establish connections between SDGs and oral health care, in addition to regulating their SDP. Stakeholders in the dentistry sector can benefit from this research by understanding how dental students now perceive the SDGs and SDP; this knowledge can then be used to develop dental policy curricula and improve sustainability competency.

This will better prepare students to serve as educators as well as professionals, aligning with their commitment to integrating the various principles and objectives of SDGs and SDP into education and practice within dental care.

Existing literature has identified a scarcity of available data related to the interconnections between oral health and the various SDGs at the country level. As far as we are aware, no study exists on how Saudi Arabian dental students consider SDGs and SDP as reflecting less sustainability in the dental care system. Therefore, this cross-sectional study was conducted to assess Saudi dental students’ self-perceived knowledge and awareness of the SDGs and the SDP. The study also explored the association between various sociodemographic groups and knowledge and awareness of SDGs and SDP.

## Materials and methods

A cross-sectional online survey was conducted by distributing an online questionnaire. The online questionnaires have been chosen due to their high response rate, convenient accessibility, and widespread distribution. The survey methodology was ethically approved by the Prince Sultan University Institutional Review Board (PRB-2022-10-0130). The survey instrument was adapted from two previously validated and published studies, modifying it for context [18, 19]. Moreover, the final survey used in this study was piloted and no comments were received. In order to obtain consent, potential participants were required to read and agree a “participation statement” before responding to the survey components.

The targeted population consisted of undergraduate dental students enroled in Saudi Arabia’s public and private dental colleges. Utilising the support of the Ministry of Education in Riyadh, Saudi Arabia, each respective college received participation requests and selected a student representative who agreed to participate, and their role was to remind and distribute surveys among the students. The questionnaires were distributed by November 2022, and data collection for this study took place over a year-long period (16 November 2022–15 November 2023). A repeated reminder was sent through the student representative. During the data collection period, approximately 1300 undergraduate dental students were enroled in Saudi Arabia.

All the data was collected through a web-based instrument, Google Forms. The survey followed a mandatory response format, requiring participants to answer all questions in order to submit their responses; however, they were free to voluntarily abandon the survey at any point. Additionally, participants were limited to providing a single response, thus allowing them to respond only once.

The questionnaire had thirty-four questions and was divided into seven sections: 1) general demographic data (three questions), 2) source of information about SDGs (one question), 3) self- reported knowledge of SDGs (three questions), 4) learning level of SDGs (eleven questions), 5) awareness of SDGs (seven questions), 6) awareness of SDP (eight questions), and 7) barriers to the implementation of SDP (one question).

The questionnaire was either closed-ended or Likert scale with must respond pattern. Closed-ended questions queried participants’ general demographic data, source of information about SDGs, learning level of SDGs and barriers to the implementation of SDP; meanwhile, Likert scale questions queried participants as to their self-reported knowledge of SDGs, awareness towards SDGs and awareness towards SDP. The five-point scale went from “not at all”, “slightly”, “moderately”, “quite”, to “extremely”.

After participants responded anonymously to the surveys, all the data was summed up for analysis. The data was analysed in different stages where the initial step included the preliminary analysis and psychometric properties of the study variables. The second step explained a descriptive analysis of each variable to explore the knowledge and awareness towards SDGs and SDP, along with subscales. The third step explained the association between different factors (gender, age and level of education) and knowledge and awareness towards SDGs and SDP. These associations were found by using the independent sample *t*-test. SPSS version 29 was used for statistical analysis, and a *p* value below 0.05 was considered significant.

## Results

### General demographics

total of 841 undergraduate dental students from different Saudi dental colleges, ranging in age from 18 to 35 years old. Therefore, the response rate was 64.7%. Both male and female students participated concurrently; however, the proportion of female (70.6%, 594 students) participants was greater than that of male (29.4%, 247 students) participants.

583 participants, which represents 69.3% of the sample, reported being between the ages of 18 and 25. In contrast, 258 individuals, representing 30.7% of the sample, fell within the age range of 26–35.

Pre-clinical students, specifically encompassing 1st and 2nd year students, accounted for 326 participants (38.8%). On the other hand, 515 participants (61.2%) reported falling under the category of clinical students, comprising 3rd, 4th, and 5th year students.

### Source of Information about SDGs

This study surveyed potential resources to impart knowledge about the SDGs among the participants. The internet was the primary source of information on the SDGs for around 59.9% of the participants. School, college, or university accounted for 10.2% of the participants, while specific training, events, or workshops related to the SDGs accounted for 4.1%. Nevertheless, a substantial portion of the participants, specifically 25.8%, possessed absolutely no knowledge related to the SDGs.

Following is a list of the four categories, ordered by the frequency and percentage of responses:

1. Internet: 504 (59.9%)
2. No knowledge: 217 (25.8%)
3. School, college, or university: 86 (10.2%)
4. Specific training, events, or workshops related to the SDGs: 34 (4.1%)

### Self-reported knowledge of SDGs

**Fig. 1:**
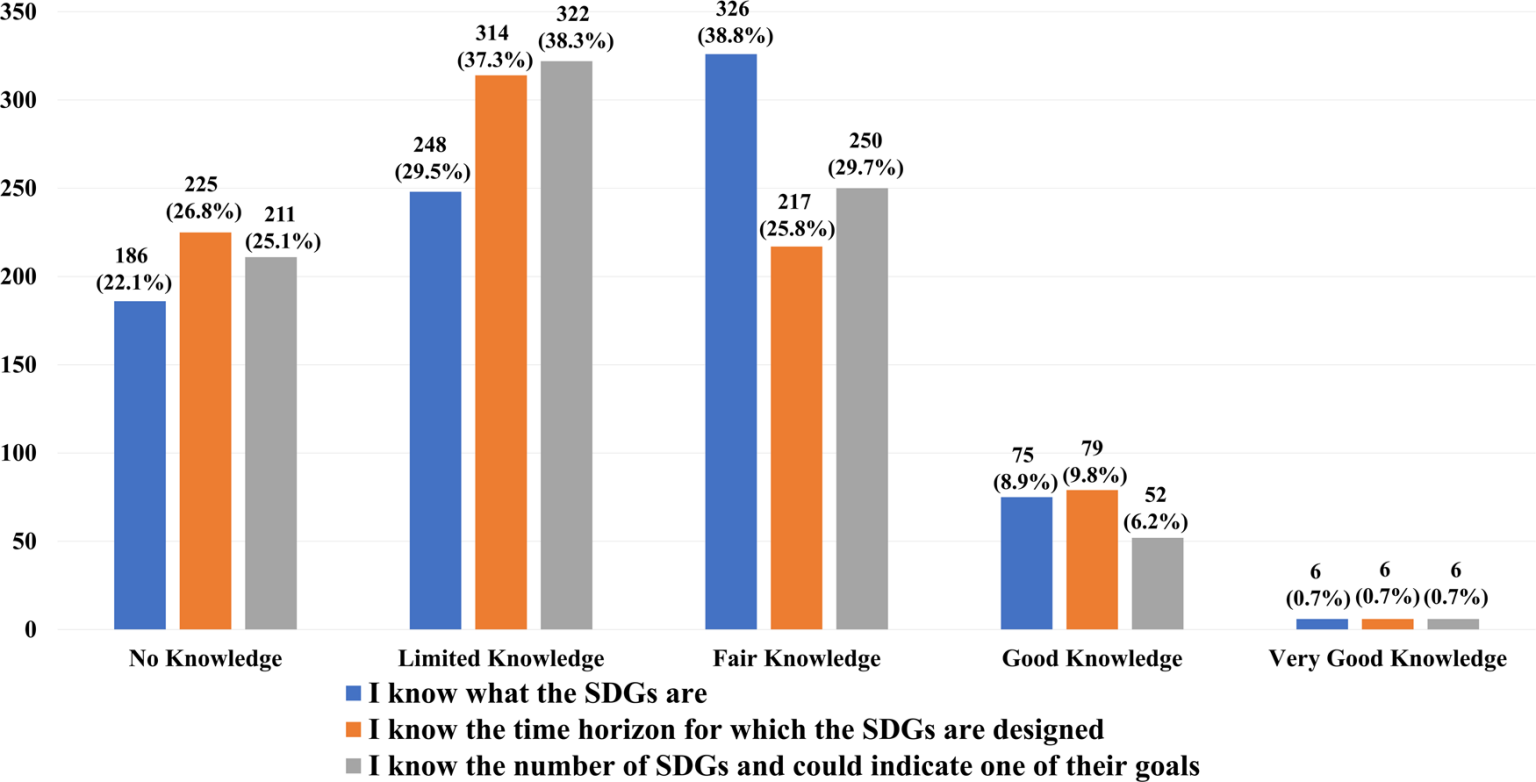
The participants’ self-reported knowledge about SDGs.

A total of three questions were asked in this section to assess the self-reported knowledge of the SDGs. Each question has five options to answer: a) no knowledge; b) limited knowledge; c) fair knowledge; d) good knowledge; and e) very good knowledge. Figure 1 shows the detailed results of the self-reported knowledge about SDGs.

### Learning level of SDGs

This part attempted to evaluate students’ basic knowledge about the SDGs by presenting a series of general questions regarding them. Eleven multiple choice questions were asked to the students, and the results are presented in Table 1.

**Table 1:** Frequencies and percentages of correct responses to different questions assessing participants’ basic SDGs knowledge.

Survey Questions	Participant’s Correct Response
1. SDGs means	784 (93.2%)
2. How many goals of SDGs	623 (74.1%)
3. How many targets of SDGs	347 (41.3%)
4. Name of organisation that launched SDGs	579 (68.8%)
5. The year the SDGs were launched	643 (76.5%)
6. The year SDGs will end	764 (90.8%)
7. Which of the following is NOT among the 5Ps of SDGs	284 (33.8%)
8. Which of the following is NOT among the three pillars of sustainable development?	661 (78.6%)
9. Which of the following is NOT a sustainable goal targeted to be achieved by 2030?	587 (69.8%)
10. Which SDG ensures sustainable management of good health and wellbeing for all?	592 (70.4%)
11. Which SDG ensures sustainable management of gender equality?	424 (50.4%)

Total participants = 841.

### Awareness of SDGs and SDP

Participants’ awareness of the SDGs and SDP was assessed through a total of seven and eight statements, respectively. The frequencies and percentages of awareness towards SDGs and SDP for all statements are shown in Table 2.

**Table 2:** Frequencies and percentages of participants’ awareness of SDGs and SDP (*N* = 841).

Survey questions	Not at all	Slightly	Moderately	Quite	Extremely
*Awareness of SDGs*					
1. I am aware of the importance of SDGs	136 (16.2%)	39 (4.6%)	531 (63.1%)	110 (13.1%)	25 (3.0%)
2. I am interested in SDGs	123 (14.6%)	64 (7.6%)	423 (50.3%)	186 (22.1%)	45 (5.4%)
3. I think SDG training is important in undergraduate education	123 (14.6%)	92 (10.9%)	391 (46.5%)	188 (22.4%)	47 (5.6%)
4. I want to learn more about SDGs	92 (10.9%)	45 (5.4%)	380 (45.2%)	250 (29.7%)	74 (8.8%)
5. I am hopeful about sustainable change	98 (11.7%)	45 (5.4%)	435 (51.7%)	212 (25.2%)	51 (6.1%)
6. I’d like to be more involved in action that helps to achieve the SDGs	105 (12.5%)	76 (9.0%)	401 (47.7%)	200 (23.8%)	59 (7.0%)
7. I think SDGs are important in future dental practice	110 (13.1%)	26 (3.1%)	250 (29.7%)	255 (30.3%)	200 (23.8%)
*Awareness of SDP*					
1. I am aware of the importance of SDP	141 (16.8%)	38 (4.5%)	55 (6.5%)	346 (41.1%)	261 (31.0%)
2. I am interested in SDP	141 (16.8%)	33 (3.9%)	49 (5.8%)	366 (43.5%)	252 (30.0%)
3. I think undergraduate training about SDP is important	126 (15.0%)	47 (5.6%)	59 (7.0%)	332 (39.5%)	277 (32.9%)
4. I want to learn more about SDP	122 (14.5%)	39 (4.6%)	43 (5.1%)	328 (39.0%)	309 (36.7%)
5. I am worried about whether or not my dental practice is sustainable	116 (13.8%)	77 (9.2%)	62 (7.4%)	333 (39.6%)	253 (30.1%)
6. The environmental impact of my dental work is important to me	128 (15.2%)	59 (7.0%)	29 (3.4%)	347 (41.3%)	278 (33.1%)
7. I am worried about the waste that dental practices produce	128 (15.2%)	43 (5.1%)	45 (5.4%)	337 (40.1%)	288 (34.2%)
8. I believe that this is the time to seriously consider the 4Rs (reduce, reuse, recycle, and rethink)	108 (12.8%)	43 (5.1%)	91 (10.8%)	251 (29.8%)	348 (41.4%)

*SDGs* Sustainable Development Goals, *SDP* Sustainable Dental Practice.

### Descriptive statistics of variables to explore the self-reported knowledge about SDGs, as well as the awareness of SDGs and SDP among dental students in Saudi Arabia

Table 3 shows descriptive statistics relating to each study variable. Participants reported a moderate level of self-reported knowledge (M = 6.76, 95% CI [6.58, 6.93]) and awareness regarding SDGs (M = 21.53, 95% CI [21.08, 21.97]). However, participants reported a high level of awareness regarding SDP (M = 29.68, 95% CI [28.96, 30.39]).

**Table 3:** Descriptive statistics for study variables (*N* = 841).

Variables	*N*	Mean (SD)	95% CI	Min	Max
Self-reported knowledge of SDGs	841	6.76 (2.60)	6.58, 6.93	3	12
Awareness of SDGs	841	21.53 (6.61)	21.08, 21.97	7	34
Awareness of SDP	841	29.68 (10.49)	28.96, 30.39	8	40

*SD* Std. Deviation, *SDGs* Sustainable Development Goals, *SDP* Sustainable Dental Practice.

### Factors associated with participants’ self-reported knowledge and awareness of SDGs as well as awareness of SDP

An independent sample *t*-test was conducted for participants’ knowledge and awareness with their demographic characteristics (gender, age, and level of education).

#### Gender

The findings indicated a significant association between gender and all the variables, including self-reported knowledge of SDGs, awareness of SDGs, and awareness of SDP (*p* < 0.05). The findings revealed that females exhibited a higher level of knowledge of SDGs and a greater awareness of both SDGs and SDP, as shown by the evidence presented in Table 4.

**Table 4:** Independent Samples *t*-test determining association between factors (gender, age, and level of education), self-reported knowledge and awareness of SDGs and awareness of SDP (*N* = 841).

Gender						
	Male		Female		*t* (839)	*p*
	*M*	*SD*	*M*	*SD*		
Knowledge of SDGs	4.72	2.32	7.61	2.20	–17.01	<0.001^***^
Awareness of SDGs	16.01	8.05	23.82	4.13	–18.50	<0.001^***^
Awareness of SDP	19.62	11.42	33.86	6.49	–22.79	<0.001^***^

Age						
	18–25 Years		26–35 Years		*t* (839)	*p*
	*M*	*SD*	*M*	*SD*		
Knowledge of SDGs	6.74	2.52	6.79	2.76	–0.21	0.82
Awareness of SDGs	21.50	6.16	21.59	7.54	–0.17	0.86
Awareness of SDP	29.90	10.30	29.18	10.93	0.91	0.36

Level of Education						
	Pre-Clinical		Clinical		*t* (839)	*p*
	*M*	*SD*	*M*	*SD*		
Knowledge of SDGs	6.84	2.60	6.70	2.60	0.76	0.44
Awareness of SDGs	21.98	6.58	21.24	6.62	1.57	0.11
Awareness of SDP	28.68	10.29	30.30	10.58	–2.18	0.02^*^

*SDGs* Sustainable Development Goals, *SDP* Sustainable Dental Practices.

*p < 0.05; **p < 0.01; ***p < 0.001.

#### Age

No significant disparities were observed between the variables and age cohorts, including knowledge of SDGs, awareness of SDGs, and awareness of SDP (*p* > 0.05) (Table 4).

#### Level of education

Participants’ level of education was not significantly related to their knowledge and awareness of SDGs (*p* > 0.05).

However, a significant association was observed between participants’ education levels and their degree of awareness of SDP. The results of this study revealed statistically significant differences in the awareness of SDP between pre-clinical and clinical students (*p* < 0.02). The findings revealed that clinical students (M = 30.30, SD = 10.58) exhibited a higher level of awareness regarding SDP in comparison to pre-clinical students (M = 28.68, SD = 10.29) (Table 4).

### Barriers to the implementation of SDP

**Fig. 2:**
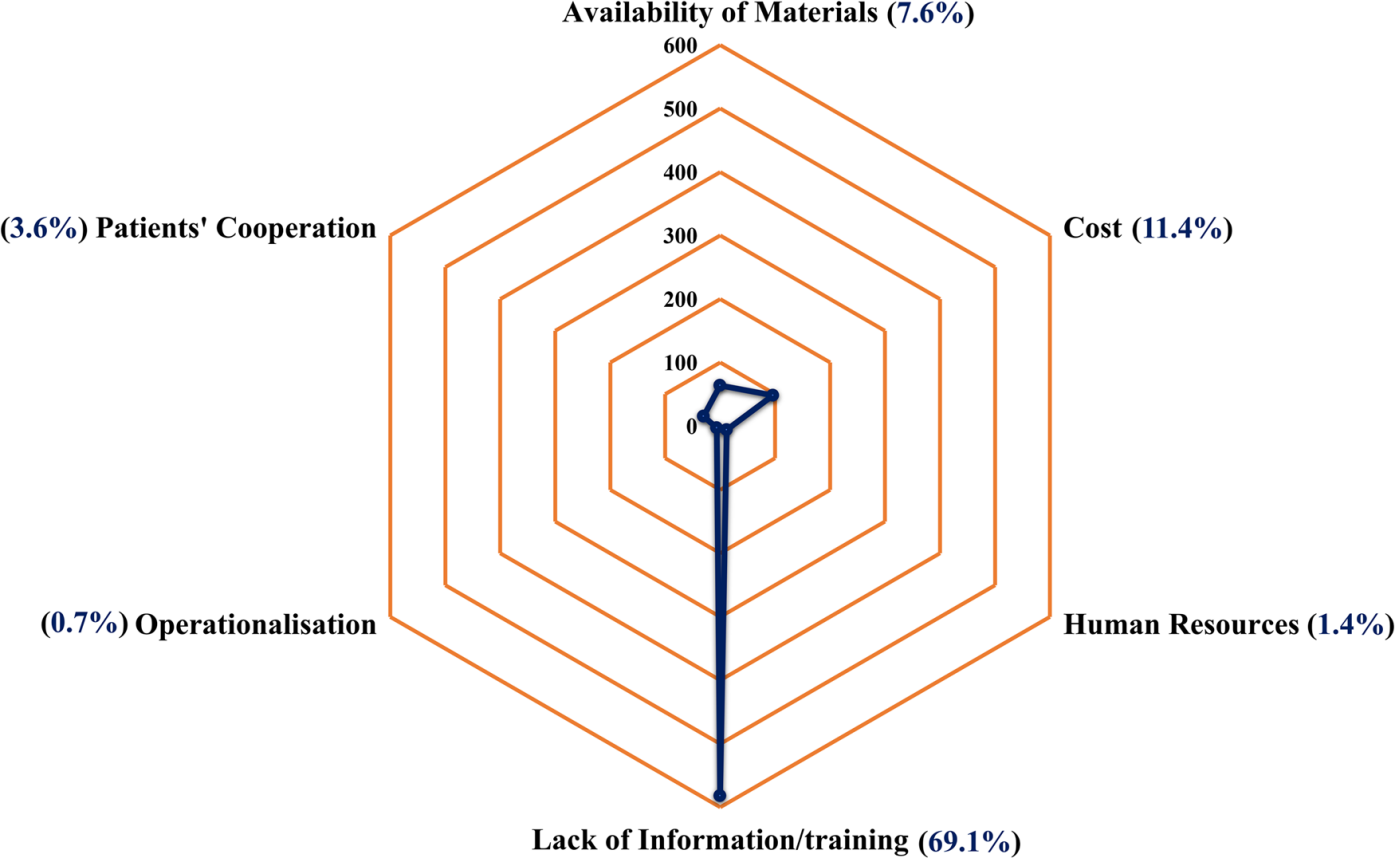
The participants’ identification of the barriers to implementing SDP.

Most participants identified a lack of information or training (69.1%) as their primary barrier to implementing SDP, followed by costs (11.4%) and the availability of materials (7.6%). Only a small percentage of participants, 3.6% and 1.4% respectively, identified patient cooperation and human resources as barriers to implementing SDP (Fig. 2).

## Discussion

The primary aim of this study was to assess the level of self-perceived knowledge among undergraduate dental students in Saudi Arabia regarding SDGs, their awareness of SDGs, as well as SDP. The present study further aimed to investigate the potential relationship between various demographic factors, including age, gender, level of education, and self-perceived knowledge of the SDGs, awareness of the SDGs, and SDP. In addition, this research study sought to identify potential barriers that may hinder the successful implementation of SDP and identify sources of information and knowledge regarding SDGs. The findings of this research study offer valuable insights into the level of knowledge and awareness among dental students in Saudi Arabia regarding SDGs and SDP.

A total of 841 Saudi dental students participated in this study, which is notably larger in comparison to most of the previous studies conducted on sustainability among dental students globally [15, 16, 18, 20–24]. Even though there are no specific rules for the breakdown of student enrolment in Saudi Arabia, the gender distribution of the participants in this study was predominantly female, aligning with the findings of previous surveys conducted in this field [24]. The emerging pattern of increased female involvement in volunteer activities suggests a potential gender disparity, indicating that female students may exhibit a greater inclination towards engaging in such activities compared to their male counterparts.

The findings of this study support the notion that a significant proportion (59.9%) of students rely on online sources. However, a noteworthy percentage (25.8%) of students exhibited no familiarity with SDGs. In contrast, only a small fraction (10.2%) of students acquired knowledge about SDGs through educational institutions. These findings imply that educational institutions have a crucial role to play in introducing supplementary measures associated with SDGs in academic settings at different levels. This approach can enhance awareness, knowledge, and involvement among a larger population of students regarding SDGs. In a Spanish survey, many university students were unfamiliar with SDGs, and few had learned about them from the internet, universities, and other sources [25]. Another Indonesian academic survey found that 54.5% of university students acquired SDG knowledge online, whereas 62% did not understand them [26].

Based on our findings, the majority of the dental students possessed a moderate level of self- reported knowledge and awareness regarding the SDGs. This finding suggests that Saudi dental students are still lacking in their knowledge and awareness of the UN’s SDGs. Bhambra [4] emphasised the importance of understanding SDGs within dental practices. She suggested dental practices can enhance the growth of dental professionals in a sustainable manner by investing in various initiatives such as continuing education opportunities, mentoring programs, and career advancement pathways. The International Dental Federation also recognizes the importance of the SDGs and emphasizes the integration of these goals into the daily practice of the dental profession, as stated in its public statement [9]. Several studies have been conducted to investigate the integration of the objectives and principles of different SDGs into dental practices and dental education. These studies have shown a strong dedication to incorporating these principles and objectives within the dental field. The acknowledgement of the significance lies in not solely delivering high-quality dental care, but also in tackling wider concerns associated with education, health, environmental sustainability, gender equality, and also social inclusion. This collective effort contributes to the overall progress of the SDGs [15, 27].

Additionally, the study investigated the level of awareness regarding SDP in addition to the awareness of SDGs. The findings revealed that the participants demonstrated an impressive level of awareness when it came to SDP. The study findings were found to be in line with prior research conducted among undergraduate dental students in the United States, which revealed that a significant majority of participants, specifically 83%, exhibited a favourable attitude towards environmental sustainability within the field of dentistry. In a recent Saudi context study conducted by Jamal et al. [21], it was found that a significant proportion of Saudi dental students (73%) and Saudi faculty members (83.6%) lacked awareness regarding environmental sustainability in dentistry. These findings, however, differ from the results obtained in the present study. In a comprehensive review conducted recently by Jalbani et al. [7], the authors reviewed 11 research papers focused on the topic of sustainability in dentistry. The findings of this study revealed that dental practitioners possess a commendable level of knowledge regarding sustainability in dentistry and exhibit positive attitudes towards environmental conservation. However, the implementation of these principles in their day-to-day practice was found to be insufficient.

Nevertheless, when the participants were asked about the barriers interfering with the successful implementation of SDP, a significant majority of 69.1% indicated a lack of information or training as the primary barrier, followed by the cost of implementation (11.4%). A study in the UK and the US showed that dental students expressed significant concern regarding their limited understanding of environmental sustainability within the field of dentistry. This lack of knowledge was identified as a primary barrier influencing their attitudes towards embracing sustainability practices in dentistry [22]. Lack of knowledge was also the prime factor that influenced the adoption of SDP in India [23]. A study involving Portuguese dental practitioners found costs to be a prime barrier, followed by a lack of information and training for the implementation of SDP [16]. Al Shatrat et al. [28] reported similar findings, indicating that costs were identified as a significant barrier to the implementation of SDP in Jordan.

The existing literature as well as the present study reveal that there is a notable presence of positive attitudes towards the adoption of sustainability in the field of dentistry, despite a lack of comprehensive sustainability knowledge among students and educators. The findings suggest a growing need for educational initiatives focused on sustainability. The incorporation of sustainability into dental education can be proposed as a means to establish enhanced legitimacy and facilitate substantial transformation. The integration of sustainable health into learning outcomes is a crucial consideration for policymakers. It is imperative for dental education providers to take on the responsibility of incorporating sustainability principles into all dental curricula.

This study additionally examined potential factors that could be associated with participants’ self-reported knowledge about SDGs and their awareness of both SDGs and SDP. The findings revealed that gender was significantly associated with participants’ self-reported knowledge of SDGs and awareness of SDGs and SDP. On the other hand, the level of education was found to be associated only with participants’ awareness of SDP.

The female dental students demonstrated a superior understanding of the SDGs and displayed a heightened awareness of both the SDGs and the SDP. The present findings align with prior research that has indicated the existence of an ‘eco gender gap’, wherein men tend to exhibit lower levels of commitment towards adopting and maintaining an environmentally friendly lifestyle compared to women. Multiple research studies have reported findings suggesting that women tend to demonstrate a higher inclination towards engaging in eco-friendly behaviours and hold more pronounced environmental values in comparison to men [24, 29].

Furthermore, clinical dental students demonstrated a greater degree of awareness regarding SDP when compared to their pre-clinical counterparts. The observed finding can be attributed to various potential explanations. It is possible that students who actively participate in clinical activities may have a better grasp of the environmental consequences associated with dental practices. For instance, their direct involvement in dental clinics, where substantial amounts of plastic waste are generated and significant quantities of water and electricity are consumed, could contribute to their increased awareness. However, it would be interesting to explore further investigation to determine the extent of the relationship between clinical students and their awareness regarding the selection of equipment and materials, energy-saving and renewable energy, disposal of waste, and biodiversity during their dental practice. In addition, another potential area for future research would involve comparing the responses of students from private and public dental colleges, a facet that was not explored in this study.

Online surveys have become increasingly popular due to their ease, convenience, and cost-effectiveness as a method of collecting data. Nevertheless, there are certain drawbacks to their widespread use. One limitation is the challenge of accurately describing the population being surveyed. Additionally, there is a risk of respondents with biases selectively choosing to participate, which can result in the spread of misinformation. Even so, it is important to recognise the advantages of the online survey since it reassures complete anonymity, allowing respondents to freely share their knowledge and attitudes.

## Conclusion

This study is the first to investigate the self-reported knowledge and awareness levels of Saudi dental students regarding the SDGs and SDP. In general, it was observed that participants possess a moderate level of self-reported knowledge of and awareness of SDGs, while demonstrating a higher level of awareness concerning SDP. The findings also indicate that female students possess a greater knowledge of SDGs and exhibit awareness of both SDGs and SDP. Additionally, it was observed that students who participate in clinical activities demonstrate a higher level of awareness regarding SDP.

In the field of dentistry, the concept of sustainability holds significant importance and encompasses a wide range of individuals and organisations involved in oral healthcare. These stakeholders include the oral health team, government bodies, scientific researchers, educators, students, manufacturers, distributors, dental equipment technicians, waste collectors, processors, and patients. It is imperative for dental professionals to place the highest priority on preventing oral diseases and promoting oral health. This approach aims to decrease the necessity for future treatments while ensuring that the safety of patients and the quality of their care are not compromised. Additionally, it is crucial to minimise the consumption of materials in this endeavour. The dental industry ought to be encouraged to foster the advancement of sustainable and environmentally friendly dental materials and technologies. It is the responsibility of governments and policymakers to encourage and promote environmentally sustainable practices. Active involvement in policymaking at all levels is essential for professionals. From practicing at home to expanding to a larger scale, whether it be within your region, country, or even globally. This is critical for developing and disseminating optimal guidelines. It is critical to carefully consider these factors, ensuring a balanced approach that considers the patient’s well-being and the impact on the environment. Patients must be well-informed during their visits to dental professionals and consider the potential negative effects of their treatment choices on the environment and the wellbeing of future generations. Through the concerted efforts of all stakeholders, a sustainable dental environment can be established that not only aligns with the SDGs, but also promotes SDP.

## Data Availability

The databases used and/or analysed during the current study are available from the corresponding author upon request.
